# Potential Osteoinductive Effects of Hydroxyapatite Nanoparticles on Mesenchymal Stem Cells by Endothelial Cell Interaction

**DOI:** 10.1186/s11671-021-03522-1

**Published:** 2021-04-26

**Authors:** Zhongyi Wang, Tianlei Han, Haoqi Zhu, Jinxin Tang, Yanyang Guo, Yabing Jin, Yu Wang, Guilan Chen, Ning Gu, Chen Wang

**Affiliations:** 1 Jiangsu Key Laboratory of Oral Diseases, Department of Prosthodontics, Affiliated Hospital of Stomatology, Nanjing Medical University, No. 136, Han-zhong Road, Nanjing, 210029 People’s Republic of China; 2 Department of Physics, City University of Hong Kong, Kowloon, Hong Kong SAR China; 3 Jiangsu Key Laboratory of Oral Diseases, Department of Laboratory Medicine, Affiliated Hospital of Stomatology, Nanjing Medical University, Nanjing, 210029 China

**Keywords:** Hydroxyapatite nanoparticles, HWJ-MSC, HUVEC, HIF-1α, ERK1/2 pathway, Two-stage cell-lineage model

## Abstract

Nano-hydroxyapatite (nano-HA) has attracted substantial attention in the field of regenerative medicine. Endothelial cell (EC)-mesenchymal stem cell (MSC) interactions are necessary for bone reconstruction, but the manner in which nano-HA interacts in this process remains unknown. Herein, we investigated the cytotoxicity and osteoinductive effects of HA nanoparticles (HANPs) on MSCs using an indirect co-culture model mediated by ECs and highlighted the underlying mechanisms. It was found that at a subcytotoxic dose, HANPs increased the viability and expression of osteoblast genes, as well as mineralized nodules and alkaline phosphatase production of MSCs. These phenomena relied on HIF-1α secreted by ECs, which triggered the ERK1/2 signaling cascade. In addition, a two-stage cell-lineage mathematical model was established to quantitatively analyze the impact of HIF-1α on the osteogenic differentiation of MSCs. It demonstrated that HIF-1α exerted a dose-dependent stimulatory effect on the osteogenic differentiation rate of MSCs up to 1500 pg/mL, which was in agreement with the above results. Our data implied that cooperative interactions between HANPs, ECs, and MSCs likely serve to stimulate bone regeneration. Furthermore, the two-stage cell-lineage model is helpful in vitro system for assessing the potential influence of effector molecules in bone tissue engineering.

**Supplementary Information:**

The online version contains supplementary material available at 10.1186/s11671-021-03522-1.

## Introduction

The reconstruction of bone defects caused by trauma, congenital malformation, or surgical resection poses a great challenge to orthopedic surgery [1]. Hydroxyapatite (HA), a representative bioactive ceramic, had been employed as a bone substitute [2]. However, undesirable mechanical and osteoinductive properties restrict its clinical application [3]. In recent years, nano-HA has demonstrated more optimal better bioactivity and improved mechanical performances due to its unique bionic characteristics and has garnered significant interest in biomedical fields related to regenerative medicine [4]. When nano-HA is implanted into bone defects, multiple cells involved in bone repair will be exposed to it. As such, it is necessary to assess the biological behavior of nano-HA. Several lines of evidence have directly demonstrated that HA nanoparticles (HANPs) can be uptaken by human umbilical cord Wharton’s jelly-derived mesenchymal stem cells (hWJ-MSCs) and osteoblast cells, resulting in enhanced osteogenic differentiation [5–7]. Dua et al. previously reported the ability of HANPs to promote the integration of engineered cartilage into de novo cartilage [8]; conversely, HANPs inhibit the angiogenic ability of human umbilical vein endothelial cells (HUVECs) [9]. In terms of human health, a more comprehensive understanding of the impact of HANPs on bone regeneration is warranted, and ongoing applications of engineered nano-based artificial bone add to the urgency of such studies.

Bone regeneration is inevitably accompanied by the invasion of neovessels. ECs are the inner cellular lining of the vascular system that serve to passively deliver blood and also play a role in inducing, specifying, and guiding organ regeneration as well as maintaining homeostasis and metabolism [10, 11]. MSCs are a part of the periendothelial niche and possess self-renewal and multi-differentiation abilities under the induction of particular physiological and biochemical microenvironments within their resident niches [12]. Tsai et al. found that ECs can secrete endothelin-1 to direct MSCs toward osteo- and chondro-lineage differentiation [13]. Additionally, Saleh et al. used microarray data analysis to identify HUVEC-secreted proteins and related crosstalk signaling pathways that interact with MSC membrane-bound receptors to enhance proliferation and osteogenic differentiation [14]. In bone tissue engineering, HANPs can come in contact with neovessels and be endocytosed by ECs, which has been shown to alter the physiologic function of these cells [9, 15]. This can also influence the surrounding osteoprogenitor cells and affect bone repair by altering paracrine signaling. However, while the direct impact of HANPs on MSCs has been explored, there is still a lack of a clear understanding as to whether HANPs can indirectly induce osteogenic differentiation of MSCs through ECs, which is essential to our understanding of the effect of HANPs in regard to bone repair.

In this study, in an effort to gain further insights into the biological effects of HANPs on the interaction between ECs and MSCs, an indirect co-culture model was established using HUVECs and hWJ-MSCs. By utilizing this system, the cytotoxicity and osteoinductive effects of HANPs on hWJ-MSCs via HUVEC-mediated paracrine signaling were assessed. To identify key factors influencing HANP-induced endothelial cell-MSC interactions, soluble factors in the supernatant from HUVECs that had been stimulated with HANPs were evaluated with an emphasis on the related mechanisms at both the gene and protein levels. The results demonstrated that hypoxia-inducible factor (HIF)-1α plays a critical role in these interactions.

To quantitatively observe and predict the impact of HIF-1α on the process of osteogenesis, a mathematical model that combines a two-stage cell lineage with HIF-1α was established. Here, by analyzing the empirical data, the two-stage cell lineage model was used to predict the MSC number and differentiation degree at any time point, as based on the defined initial cell seeding density and HIF-1α concentration, and this may in turn provide appropriate suggestions for initial culture conditions and incubation times. The results of this study will help shed light on the interactions between nano-based bone substitutes and biological systems, which can serve to promote the development of innovative biomaterials for use in regenerative medicine.

## Materials and Methods

### Preparation and Characterization of Particles

HANPs at 20 nm (np20), 20*80 nm (np80) and micro-sized HA particles (m-HAP) with ≥ 99.0% purity were purchased from the Nanjing Emperor Nano Material Company Ltd (Nanjing, China). The size and shape of particles were viewed using transmission electron microscopy (TEM; FEI Tecnai G2 Spirit Bio-Twin, FEI, Hillsboro, OR, USA) and scanning electron microscopy (SEM; LEO1530VP, Germany). The hydrodynamic size and zeta potential of HA particles (HAPs) were determined via Zetasizer Nano ZS90 and Mastersizer 3000 (Malvern Instruments, Malvern, UK).

### Cell Preparation and Culture

All experimental protocols were approved by the Ethics Committee of Nanjing Medical University. HUVECs and hWJ-MSCs were harvested from fresh human umbilical cords, as previously described, after achieving written informed consent from the donors [16, 17]. Briefly, the umbilical cord and umbilical vein were rinsed with phosphate-buffered saline (PBS) containing 1% penicillin and streptomycin (PS; Hyclone, GE Healthcare Life Sciences, Pasching, Austria). Then, the umbilical vein was filled with 0.1% collagenase I (Sigma, St. Louis, MO, USA) and incubated for 15 min at 37 °C. After collection, the HUVECs were cultured in EC medium (ECM) (Sciencell, San Diego, CA, USA).

Subsequently, the blood vessels were removed, and the Wharton’s jelly was cut into 1 mm^2^ pieces and then placed in 25 cm^2^ tissue culture flasks (Corning Incorporated, Corning, NY, USA). These cells were incubated in L-DMEM (GIBCO Life Technology, Grand Island, NY, USA) supplemented with 10% fetal bovine serum (GIBCO) and 1% PS.

hWJ-MSCs were assessed to confirm the phenotype using monoclonal antibodies toward CD13, CD29, CD34, CD44, CD45, CD51, and CD105 (BD Biosciences, San Jose, CA, USA). HUVECs were assessed using von Willebrand factor (vWF; Shanghai ChangDao Biotech Co, Ltd., Shanghai China). HUVECs between passages 3–7 and hWJ-MSCs between passages 3–5 were used in these experiments.

**Fig. 1:**
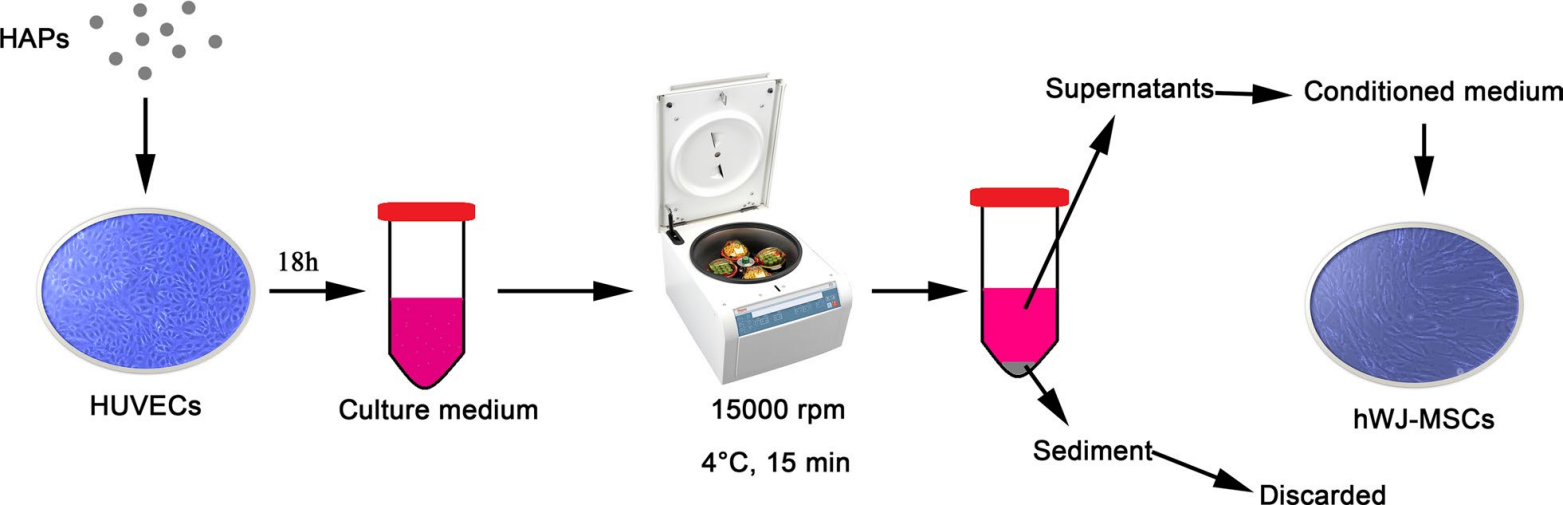
Illustration of HAPs/hWJ-MSCs indirect co-culture mediated by HUVECs. Abbreviations: *HAPs* hydroxyapatite particles, *hWJ-MSCs* human umbilical cord Wharton’s jelly-derived mesenchymal stem cells, *HUVECs* human umbilical vein endothelial cells

Particle suspensions at 1 mg/mL in PBS were then diluted in L-DMEM to the final concentration. As shown in Fig. 1, the HUVECs were incubated in the indicated concentration of HAPs for 18 h. The culture medium was centrifuged at 15,000 rpm at 4 °C for 15 min, and the supernatants supplemented with 10% FBS were used as the conditioned medium (CM) for hWJ-MSCs to accomplish the following experiments. The CM consisted of osteogenic medium supplemented with osteogenic induction fluid, which contained 10 mM β-glycerophosphate, 50 μg/mL L-ascorbic acid-2-phosphate, and 0.1 μM dexamethasone (Sigma-Aldrich, St Louis, MO, USA). Additionally, 2-methoxyestradiol (2-MeOE2) (Selleck Chemicals, Houston, TX, USA) was used as a specific HIF-1α inhibitor. In the 2-MeOE2(+) group, hWJ-MSCs were cultured with CM from HUVECs, which were pretreated with 5 μM 2-MeOE2 for 40 min before HAP supplementation. PD98059 was used as the specific MEK inhibitor. In the PD98059 group, hWJ-MSCs were cultured with CM containing 5 μM PD98059.

### Determining Cell Viability and Number

Cell viability was assessed using MTS Assay Kit (MTS; Bestbio, Beyotime Biotechnology, Shanghai, China). hWJ-MSCs were left to adhere for 24 h and then cultured with CM for 24 and 72 h. The absorbance of formazan was evaluated at 490 nm using a microplate reader (SpectraMax M2; Molecular Devices LLC, Sunnyvale, CA, USA). The absorbance was also converted to cell numbers using standard calibration curves under the same conditions.

### Quantitative Real-Time Polymerase Chain Reaction (RT-PCR)

TRIzol reagent (Invitrogen, Carlsbad, CA, USA) was used to isolate the total RNA from hWJ-MSCs cells incubated in CM for the indicated time. Complementary DNA was transcribed from 1.0 µg RNA using a PrimeScript First Strand cDNA Synthesis kit (TaKaRa, Tokyo, Japan) in a T3 thermocycler (Mastercycler 5333; Eppendorf, Hamburg, Germany). The expression levels of the indicated genes were analyzed using FastStart Universal SYBR Green Master (ROX) kit (Roche, Basel, Switzerland) on a quantitative real-time amplification system (7900HT Fast; Applied Biosystems, Foster City, CA, USA). The relative mRNA expression of the target gene was normalized to GAPDH and then determined using the $2^{{ - \Delta \Delta C_{t} }}$ method. Primer sequences for the target genes are listed in Table 1.

**Table 1:** Primers used for real-time RT-PCR

Target gene	Forward primer sequence (5′–3′)	Reverse primer sequence (5′–3′)
RUNX-2	TGGTTACTGTCATGGCGGGTA	TCTCAGATCGTTGAACCTTGCTA
Col I	TGGTGGAGCAGCAAGAGCAA	CAGTGGACAGTAGACGGAGGAAA
OCN	CACTCCTCGCCCTATTGGC	CCCTCCTGCTTGGACACAAAG
GAPDH	CTGGTAAAGTGGATATTGTTGCCAT	TGGAATCATATTGGAACATGTAAACC
ALP	GGACATGCAGTACGAGCTGA	GTCAATTCTGCCTCCTTCCA
NANOG	CAGAAGGCCTCAGCACCTAC	ATTGTTCCAGGTCTGGTTGC
OCT3/4	GTGGAGAGCAACTCCGATG	TGCAGAGCTTTGATGTCCTG
SOX2	CCAAGACGCTCATGAAGAAG	TGGTCATGGAGTTGTACTGC
GAPDH	CTGGTAAAGTGGATATTGTTGCCAT	TGGAATCATATTGGAACATGTAAACC

*RUNX-2* runt-related transcription factor 2, *Col I* type I collagen, *OCN* osteocalcin, *ALP* alkaline phosphatase, *SOX 2* SRY-related HMG-box 2

### Alizarin Red S (ARS) Staining and Quantitative Analysis

hWJ-MSCs were cultured in 12-well plates with osteogenic medium for up to 14 d, and then, extracellular matrix mineralization was observed using ARS staining (Leagene, Leagene Biotechnology, Beijing, China) after the. Briefly, the samples were fixed with absolute ethyl alcohol for 15 min and then stained in 1% (w/v) ARS (pH, 4.2) at room temperature for 5 min. The stained cells were washed twice with double-distilled water and then photographed. For quantitative analysis of the mineralization process, 300 μL of 10% (w/v) cetylpyridinium chloride monohydrate (BOMEI, BOMEI Biotechnology, Hefei, China) was added to each well, and the plates were incubated for 30 min. A total of 90 μL from each sample was transferred to a 96-well plate, and the absorbance was then measured at 405 nm in triplicate.

### Alkaline Phosphatase (ALP) Staining and Quantitative Analysis

After the hWJ-MSCs were cultured in 12-well plates with osteogenic medium for up to 14 days, ALP staining was conducted using a BCIP/NBT Alkaline Phosphatase Color Development Kit (Beyotime). Briefly, hWJ-MSCs were fixed in 4% paraformaldehyde. Then, samples were stained in a mixture of nitro-blue tetrazolium and 5-bromo-4-chloro-3-indolyl-phosphate for 4 h and photographed. To quantify ALP synthesis, cells were lysed in icy RIPA lysis buffer (Beyotime) for 30 min. Cell lysates were centrifuged at 12,000 rpm at 4 °C for 10 min, and the supernatants underwent ALP quantitative analysis using an ALP Assay kit (Beyotime). The absorbance was measured in triplicate at 405 nm and converted to ALP activity using a standard curve.

### Enzyme-Linked Immunosorbent Assay (ELISA)

HUVECs were seeded at 2 × 10^5^ cells/well. CM was collected from HUVECs cultured with HAPs for 18 h (Additional file 1), and the supernatant was subjected to ELISA analysis (Fig. 1). A human HIF-1α ELISA kit (Anhui Joyee Biotechnics, Anhui, China) was used according to the manufacturer’s instructions.

### Immunofluorescence

HUVECs were seeded on slides in 12-well plates (7.6 × 10^4^ cells/well). After exposure to CM for 18 h, cells were fixed in 4% paraformaldehyde (Biosharp, Beijing, China) and permeabilized with 0.1% Triton X‐100 (Beyotime) in PBS prior to incubation with 1% anti-HIF-1α antibody [EPR16897](ab179483, Abcam, UK) overnight at 4 ℃. Subsequently, the cells were incubated with 1% CoraLite594—conjugated Goat Anti-Rabbit IgG(H + L) (Proteintech, USA) in the dark for 1 h. Then, the nuclei were dyed with DAPI (Beyotime, Shanghai, China), which was added to the cells and reacted for 30 s. Samples were examined using a laser confocal microscope (Olympus, Japan). The fluorescence intensity was quantified using ImageJ v.1.4 analysis software (Bethesda, MD, USA).

### Western Blot Analysis

hWJ-MSCs were incubated in CM for 24 h (extracellular signal-regulated kinase (ERK)1/2, p-ERK1/2) or in osteogenic medium for 7 d (runt-related transcription factor (RUNX)-2, type 1 collagen/collagen 1 (COL I)). Then, the cells were lysed in RIPA lysis buffer for 30 min. Cell lysates were centrifuged, and the supernatants were stored at − 20 °C for analysis. After 12% SDS-PAGE, the proteins were transferred to a polyvinylidene difluoride (PVDF) membrane. The primary antibodies used were anti-p-ERK1/2, anti-ERK1/2, anti-RUNX-2, anti-COL I, and GAPDH (1:1,000, rabbit polyclonal antibodies; Cell Signaling Technology, Boston, MA, USA). After removing unbound antibodies, the membrane was incubated with secondary antibodies for 1 h. The signal on the membranes was detected using a chemiluminescence gel imaging system (LAS4000M; GE Healthcare Biosciences AB, Uppsala, Sweden). The ratio of p-ERK to ERK and RUNX-2/COL I to GAPDH was quantified using ImageJ v.1.4 analysis software (Bethesda).

### Assessment of Cell Apoptosis

hWJ-MSCs were seeded at a density of 10^5^ cells per well in 6-well plates. Adherent cells were treated with the indicated concentrations of HIF-1α for the indicated time. Cells were then collected and labeled with FITC-Annexin V and PI (Fcmacs, Nanjing, China) for 15 min in the dark. All samples were tested using a FACScan flow cytometer (BD Bioscience, San Jose, CA, USA). The data were analyzed using FlowJo v10 (BD Biosciences).

### Two-Stage Cell-Lineage Model

In consideration of the enormous potential of HAPs, and the difficulty in analyzing the co-culture system, a mathematical model was required that could provide quantitative analysis and trusted prediction was required to understand the role of HIF-1α in the osteogenic differentiation of hWJ-MSCs.

hWJ-MSCs were cultured with 0, 300, 500, 1000, 1500, 2000, 3000, and 4000 pg/mL HIF-1α as well as osteogenic induction fluid. After fitting the data at these concentrations, we use the concentrations of HIF-1α (produced by HUVECs) in the control, m-HAP, np80, and np20 groups (240, 300, 325, and 375 pg/mL, respectively) to test the fitting equations using MATLAB (MathWorks, Natick, MA, USA). In order to simplify the model, we considered their similar growth patterns at the different concentrations of HIF-1α to be identical. The average differentiation degree was employed to fit the differentiation degree-time equation. The proliferation rate, apoptosis rate, and osteogenic differentiation degrees of the hWJ-MSCs in the different groups were detected at defined times.

A simplified two-stage cell-lineage model, which was similar to a multi-stage cell-lineage model [18, 19], was established according to the experimental data. *C*_0_ and *C*_1_ represent the cell number of the hWJ-MSCs and terminal cells, respectively. *C*_0_ and *C*_1_ are governed by:

$$\left\{ \begin{gathered} \frac{{{\text{d}}C_{0} }}{{{\text{d}}t}} = \left[ {\frac{{K - C_{0} - C_{1} }}{K}p - (p - 1)} \right]\upsilon_{0} C_{0} \hfill \\ \frac{{{\text{d}}C_{1} }}{{{\text{d}}t}} = \left( {2 - \frac{{K - C_{0} - C_{1} }}{K}p - p} \right)\upsilon_{0} C_{0} - AC_{1} \hfill \\ \end{gathered} \right.$$

Here, *p*, affected by HIF-1α and time, represents the replication probability of the hWJ-MSCs. Correspondingly, *d* = 1 − *p* is the differentiation rate that can be obtained by fitting the estimated and experimental data of cell number. The parameter v_0_ quantifies how rapidly the cells divide at each lineage stage (in particular, *v* = ln2/*c*, where c is the duration of a cell cycle). The apoptosis rate of the terminal cells is symbolized by *A*. For simplicity, we neglected that the apoptosis rate will vary slightly over time, and therefore, *A* = 4.5% is a constant. *K* denotes the environmental capacity because the cells could not undergo unlimited proliferation [20]. HIF-1α increases the differentiation rate of the hWJ-MSCs, leading to a differentiation rate modeled by:

$$\begin{aligned} d & = \frac{{d_{0} }}{{1 + (r*H)^{m} }} \\ p & = 1 - d \\ \end{aligned}$$

Herein, *H* represents the concentration of HIF-1α; *d*_0_ denotes the differentiation rate at 0 pg/mL HIF-1α; r represents the intensity of regulation (herein, representing the intensity of regulation of HIF-1α on MSCs); and m corresponds to the Hill coefficient [21], scilicet the relationship between MSC differentiation rate and HIF-1α concentration.

### Statistical Analysis

All data that meet normality and homoscedasticity requirements are expressed as the mean ± standard deviation (SD) from three or more independent experiments. SPSS 24.0 software (SPSS Inc., Chicago, USA) was used to perform the statistical analyses via one-way ANOVA or two-way ANONA. A *P *value < 0.05 was considered statistically significant. Statistical analysis is presented using GraphPad Prism 5 (GraphPad Software, La Jolla, CA).

## Results

### Characterization of HAPs

**Fig. 2:**
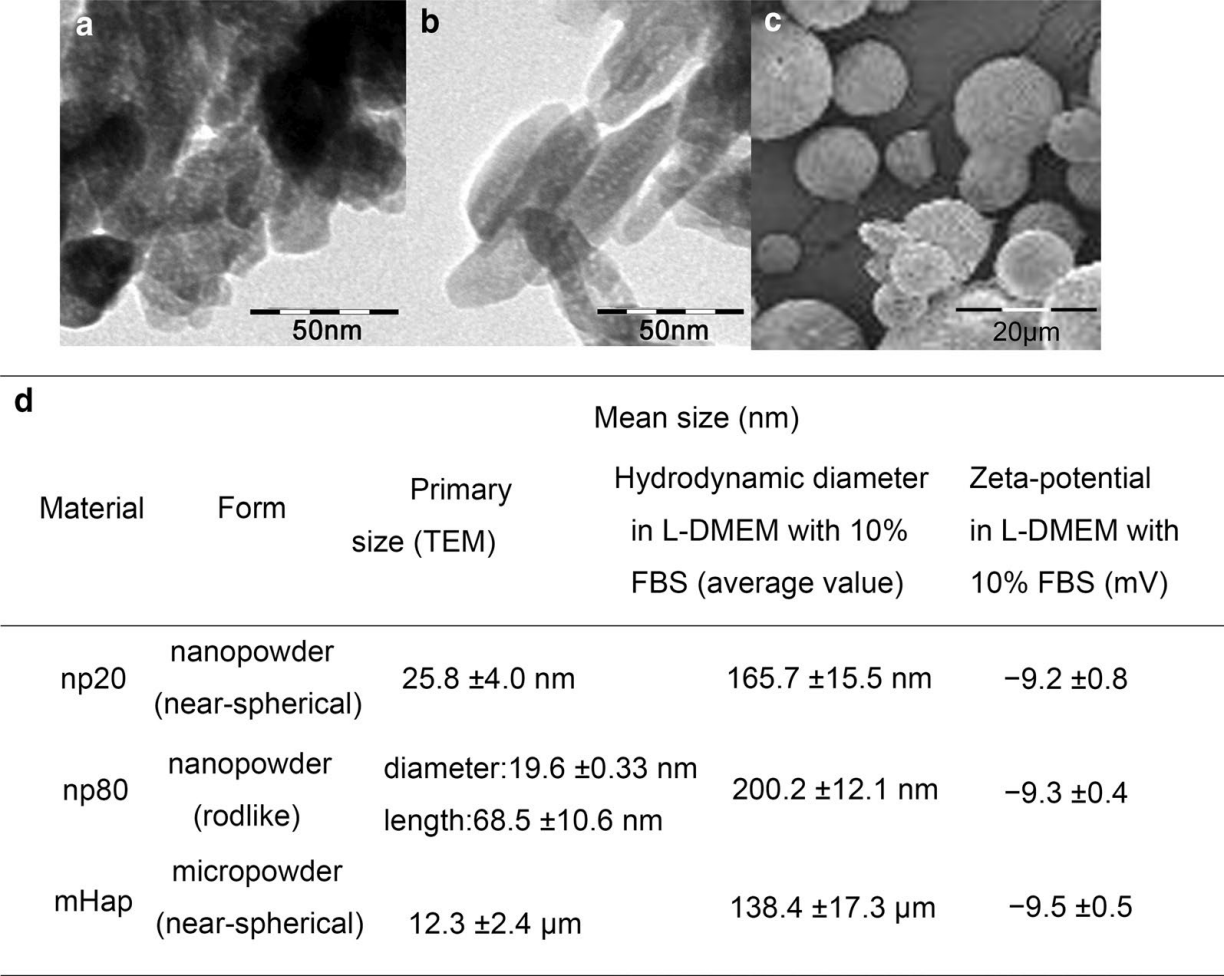
Characterization of HAPs. TEM micrographs of **a** np20 and **b** np80, and SEM micrograph of **c** m-HAP. **d** Characterization of HAPs (*n* = 6). Abbreviations: *TEM* transmission electron microscopy, *SEM* scanning electron microscopy, *HA* hydroxyapatite, *m-HAP* micro-sized HAP particles

As shown in Fig. 2, HAPs were prepared with particular size and shape. The diameters of the np20 with near-spherical shape were 20 nm on average, and the np80 was rod-like with an average length of 80 nm and width of 20 nm. The m-HAPs were also nearly spherical in shape and approximately 12 μm in diameter. All particles had a negative surface charge in L-DMEM. It has been suggested that negative values of zeta potential have a significant favorable effect on the attachment and proliferation of bone cells, as well as the direct bone bonding and new bone formation [22, 23]. Particles, which were observed in L-DMEM, have a tendency to aggregate in aqueous systems. Their hydrodynamic size was also tested, which might also be an important factor affecting their biological behaviors.

### Indirect Toxicity of HAPs Toward hWJ-MSCs

**Fig. 3:**
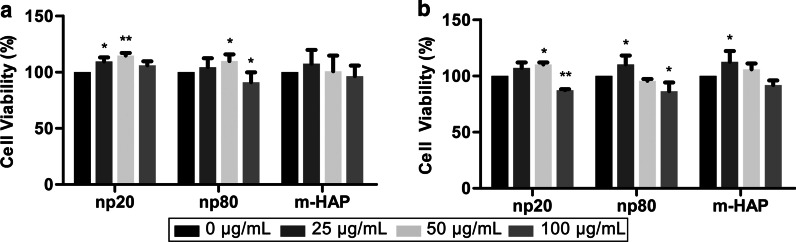
Indirect toxicity of HAPs toward hWJ-MSCs. The viability of hWJ-MSCs indirectly cultured with HAPs was measured for **a** 24 and **b** 72 h. *P < 0.05; **P < 0.01 versus the control. The control group consisted of cells incubated in CM without HAPs treatment, and cell viability was normalized as a percentage of the control. Abbreviations: *HAPs* hydroxyapatite particles, *m-HAP* micro-sized HAP particles, *hWJ-MSCs* human umbilical cord Wharton’s jelly-derived mesenchymal stem cells

To assess the indirect toxicity of HAPs on hWJ-MSCs, cell viability was measured via MTS assays. CM with 50 µg/mL HANPs could significantly stimulate hWJ-MSC viability after 24 h and 72 h and especially at 24 h. However, CM with 100 µg/mL HANPs decreased cell viability by 15–20% compared to the control after 72 h. Moreover, CM with 25 µg/mL np20, but not np80, stimulated cell viability after 24 h. These phenomena confirmed that 50 µg/mL HANPs were a subcytotoxic concentration that was used in all subsequent experiments (Fig. 3).

### Indirect Osteoinductive Effect of HAPs on hWJ-MSCs

**Fig. 4:**
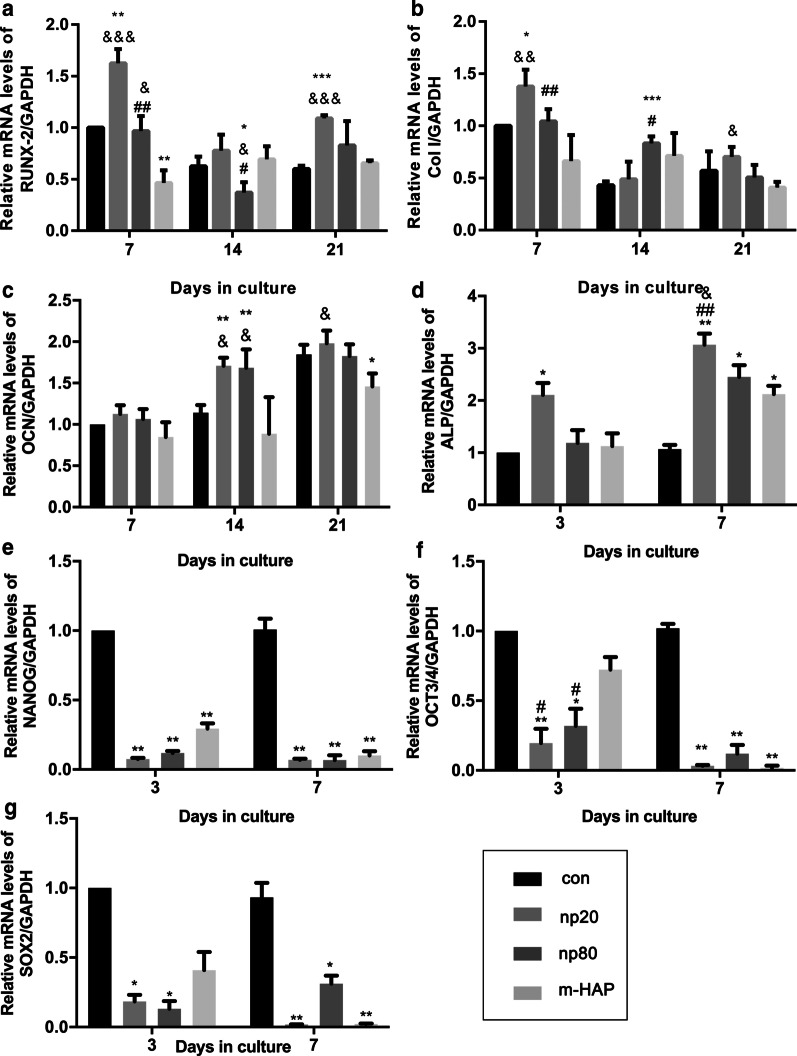
Indirect effects of HAPs on the expression of osteogenic differentiation-related genes. **a** RUNX-2, **b** Col I, **c** OCN, **d** ALP, **e** NANOG, **f** OCT3/4, and **g** SOX2 gene levels in hWJ-MSCs cultured with CM for the indicated time. *P < 0.05; **P < 0.01; ***P < 0.001 versus control group; ^&^*P* < 0.05; ^&&^*P* < 0.01; ^&&&^*P* < 0.001 versus the m-HAP group; ^#^*P* < 0.05; ^##^*P* < 0.01 versus the np20 group. Cells incubated in osteogenic medium without HAPs treatment were used as the control group. Abbreviations: *HAPs* hydroxyapatite particles, *m-HAP* micro-sized HAP particles, *hWJ-MSCs* human umbilical cord Wharton’s jelly-derived mesenchymal stem cells, *RUNX-2* runt-related transcription factor 2, *Col I* type I collagen, *OCN* osteocalcin, *ALP* alkaline phosphatase, *SOX 2* SRY-related HMG-box 2

**Fig. 5:**
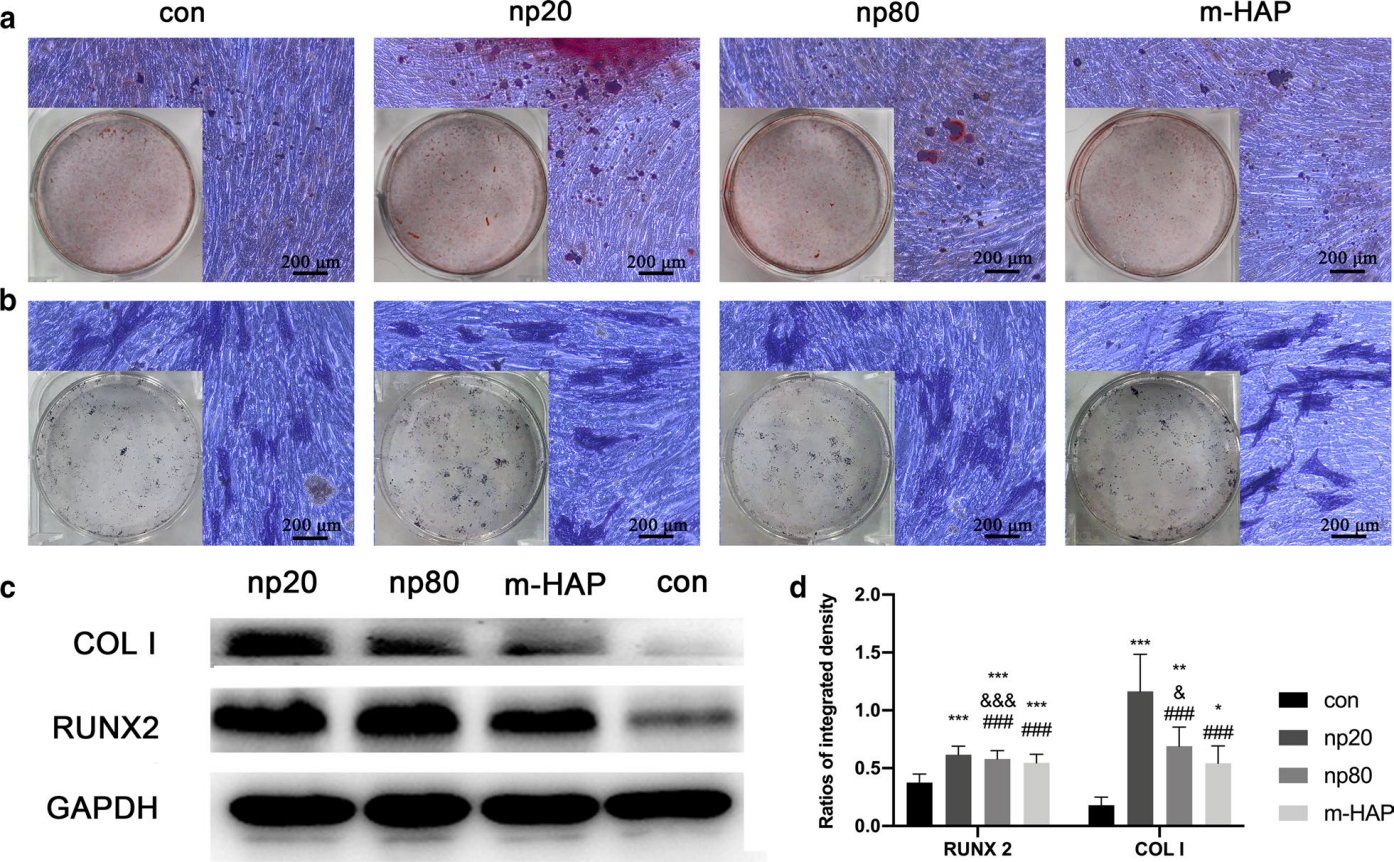
Indirect effect of HAPs on extracellular calcium deposition and ALP activity. hWJ-MSCs were incubated in osteogenic medium for 14 d. **a** Extracellular calcium deposition was then visualized via ARS staining; **b** ALP activity of the hWJ-MSCs was assessed via ALP staining, scale bars: 200 μm. **c** Western blot analysis indicated the expression of RUNX-2 and COL I of hWJ-MSCs in osteogenic medium on day 7. **d** Densitometric measurements of RUNX-2 and COL I from part (**c**). Cells incubated in osteogenic medium without HAPs treatment were used as the control group. *P < 0.05; **P < 0.01; ***P < 0.001 versus control group; ^&^*P* < 0.05; ^&&&^*P* < 0.001 versus the m-HAP group; ^###^*P* < 0.001 versus the np20 group. Abbreviations: *HAPs* hydroxyapatite particles, *m-HAP* micro-sized HAP particles, *hWJ-MSCs* human umbilical cord Wharton’s jelly-derived mesenchymal stem cells, *ALP* alkaline phosphatase

To identify the indirect osteoinductive effects of HANPs on hWJ-MSCs, the expression of osteogenic genes was assessed by quantitative RT-PCR analysis. The transcript level for runt-related transcription factor 2 (RUNX-2) in the HANP groups, especially np20, exhibited a notable increase from Day 7 to Day 21 (Fig. 4a). The gene expression of type I collagen (Col I) in the np20 group demonstrated an enhancement from Day 7 to Day 21, while the np80 group demonstrated a sustained increase from Day 7 to Day 14 (Fig. 4b). Osteocalcin (OCN) mRNA was clearly upregulated in the HANP groups on Day 14, indicating an accelerated rate of osteogenesis (Fig. 4c). The mRNA levels of alkaline phosphatase (ALP) obviously increased in the HANP groups (Fig. 4d), indicating the osteogenic differentiation of hWJ-MSCs. However, the m-HAP group presented limited changes in regard to the levels of these three osteogenic genes compared to the control group (Fig. 4). In addition, the expression of pluripotency markers, NANOG, OCT3/4, and SOX2, decreased in the HANPs groups compared with the control (Fig. 4e–g), implying that the hWJ-MSCs in the HANPs groups had differentiated, especially in the np20 group. Similar results were obtained by Western blot analysis (Fig. 5c, d), indicating that the np 20 group could indirectly enhance the expression of RUNX-2 and COL I in hWJ-MSCs.

To visually observe the indirect osteoinductive effect of HANPs on hWJ-MSCs, the cells were incubated with the indicated osteogenic medium for 14 d, followed by ARS and ALP staining. As shown in Fig. 5a, b, an increased number of mineralized nodules and higher ALP activity of the hWJ-MSCs were observed in the HANP groups compared with the m-HAP and control groups. Additionally, m-HAP, similar to the control, demonstrated limited effects on the osteogenic differentiation of the hWJ-MSCs.

### HAPs Activated ERK1/2 Signaling in hWJ-MSCs Indirectly Co-cultured with HUVECs

**Fig. 6:**
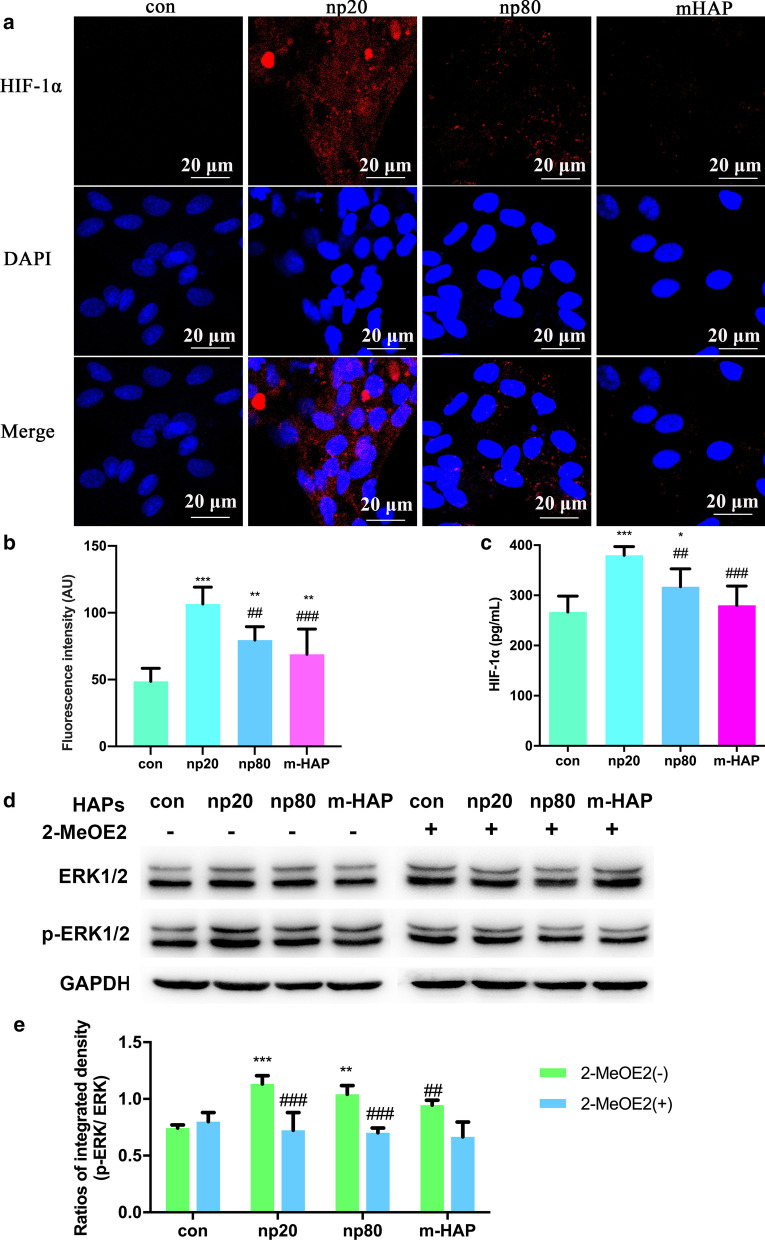
HAPs activated ERK1/2 signaling in hWJ-MSCs indirectly co-cultured with HUVECs. **a** Immunofluorescence of HIF-1α performed in HUVECs treated with/without HAPs for 18 h. **b** The fluorescence intensity of HIF-1α from part (**a**). **c** The extracellular concentration of HIF-1α in the culture medium of HUVECs treated with/without HAPs for 18 h was measured via ELISA. Scale bars: 20 μm. *P < 0.05; **P < 0.01; ***P < 0.001 versus control; ^##^*P* < 0.01; ^###^*P* < 0.001 versus the np20 group. Cells without HAPs treatment were used as the control group. hWJ-MSCs were treated with CM for 24 h. **d** Western blot analysis indicating activation of key kinases in the ERK1/2 pathways. **e** Densitometric measurements of p-ERK1/2 from part (**b**). **P < 0.01; ***P < 0.001 versus control; ^##^*P* < 0.01, ^###^*P* < 0.001 versus 2-MeOE2( −) group. Cells incubated in CM without HAP treatment were used as the control group. Abbreviations: *HAPs* hydroxyapatite particles, *m-HAP* micro-sized HAP particles, *hWJ-MSCs* human umbilical cord Wharton’s jelly-derived mesenchymal stem cells, *HUVECs* human umbilical vein endothelial cells, *ERK* extracellular signal-regulated kinase, *HIF-1α* hypoxia-inducible factor 1α

To investigate the effects on the paracrine function of HUVECs by HAPs, immunofluorescence and ELISA assays were used to identify the possible protein promoting the osteogenic differentiation of the hWJ-MSCs. As shown in Fig. 6a–c, the intracellular and extracellular production of HIF-1α was significantly facilitated by HANPs (especially np20), while there was a limited effect of m-HAP on its production.

To more accurately understand the differentiation signaling pathway of the hWJ-MSCs activated by HIF-1α, we examined the key regulators of the ERK1/2 pathway via Western blot analysis. As shown in Fig. 6d, e, while the protein levels of total ERK1/2 remained unaltered, p-ERK1/2 levels were distinctly increased in the hWJ-MSCs cultured with HANPs, and this was especially true in the np20 group. However, m-HAP had little effect on p-ERK1/2 levels in the hWJ-MSCs, similar to its effect on HIF-1α production in HUVECs. Importantly, the increased levels of p-ERK1/2 in the hWJ-MSCs activated by HIF-1α could be blocked by 2-MeOE2, a specific HIF-1α inhibitor, indicating that HIF-1α functioned upstream of the ERK1/2 signaling pathway in hWJ-MSCs.

### HIF-1α Promoted Osteogenic Differentiation of hWJ-MSCs via the ERK1/2 Pathway

**Fig. 7:**
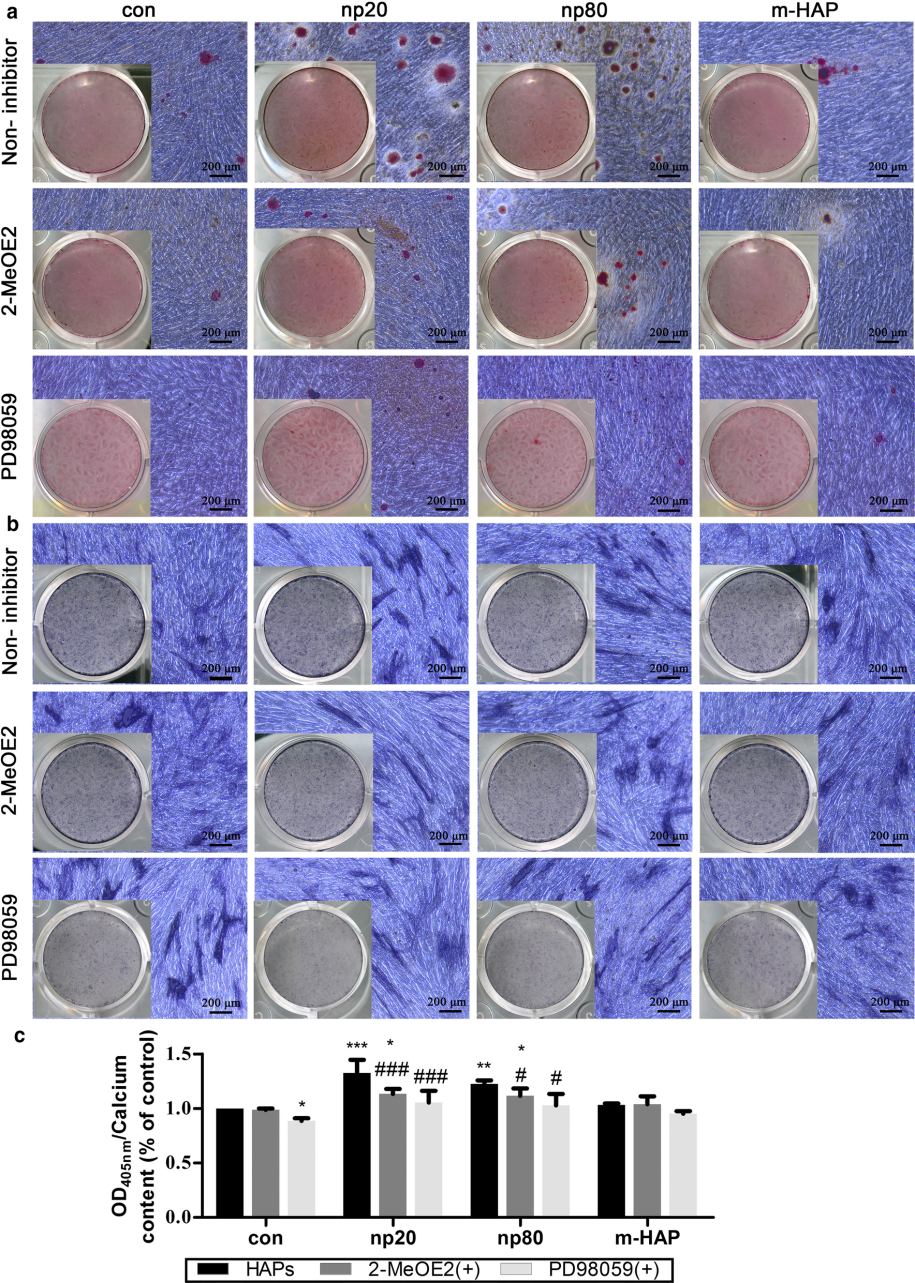
HIF-1α promoted osteogenic differentiation of hWJ-MSCs via the ERK1/2 pathway. hWJ-MSCs were incubated in osteogenic medium with or without PD98059 for 14 d. **a** Extracellular calcium deposition was visualized via ARS staining. **b** ALP activity of hWJ-MSCs was assessed via ALP staining. Scale bars: 200 μm. **c** Quantitative analysis of extracellular calcium matrix. *P < 0.05; **P < 0.01; ***P < 0.001 versus control; ^#^*P* < 0.05; ^###^*P* < 0.001 versus the np20 group. Cells incubated in osteogenic medium without HAPs and PD98059 treatment were used as the control group. Abbreviations: *HAPs* hydroxyapatite particles, *m-HAP* micro-sized HAP particles, *hWJ-MSCs* human umbilical cord Wharton’s jelly-derived mesenchymal stem cells, *HUVECs* human umbilical vein endothelial cells, *ERK* extracellular signal-regulated kinase, *HIF-1α* hypoxia-inducible factor 1α, *ALP* alkaline phosphatase

To determine whether HIF-1α was necessary for the observed stimulation of hWJ-MSC osteogenic differentiation, a specific HIF-1α inhibitor (2-MeOE2) was applied to these cell cultures. As shown in Fig. 7, the mineralized matrix deposition and ALP activity of the 2-MeOE2 (+)-treated group hWJ-MSCs cultured in osteogenic medium were weakened, indicating that HIF-1α was indispensable for the osteogenic differentiation of the hWJ-MSCs. Based on these results, we further explored the role of the ERK1/2 pathway in the osteogenic differentiation of the hWJ-MSCs activated by HIF-1α. The mineralized matrix deposition and ALP activity in the hWJ-MSCs cultured with osteogenic medium were suppressed following the administration of PD98059, a specific MEK inhibitor.

### Two-Stage Cell-Lineage Models

**Fig. 8:**
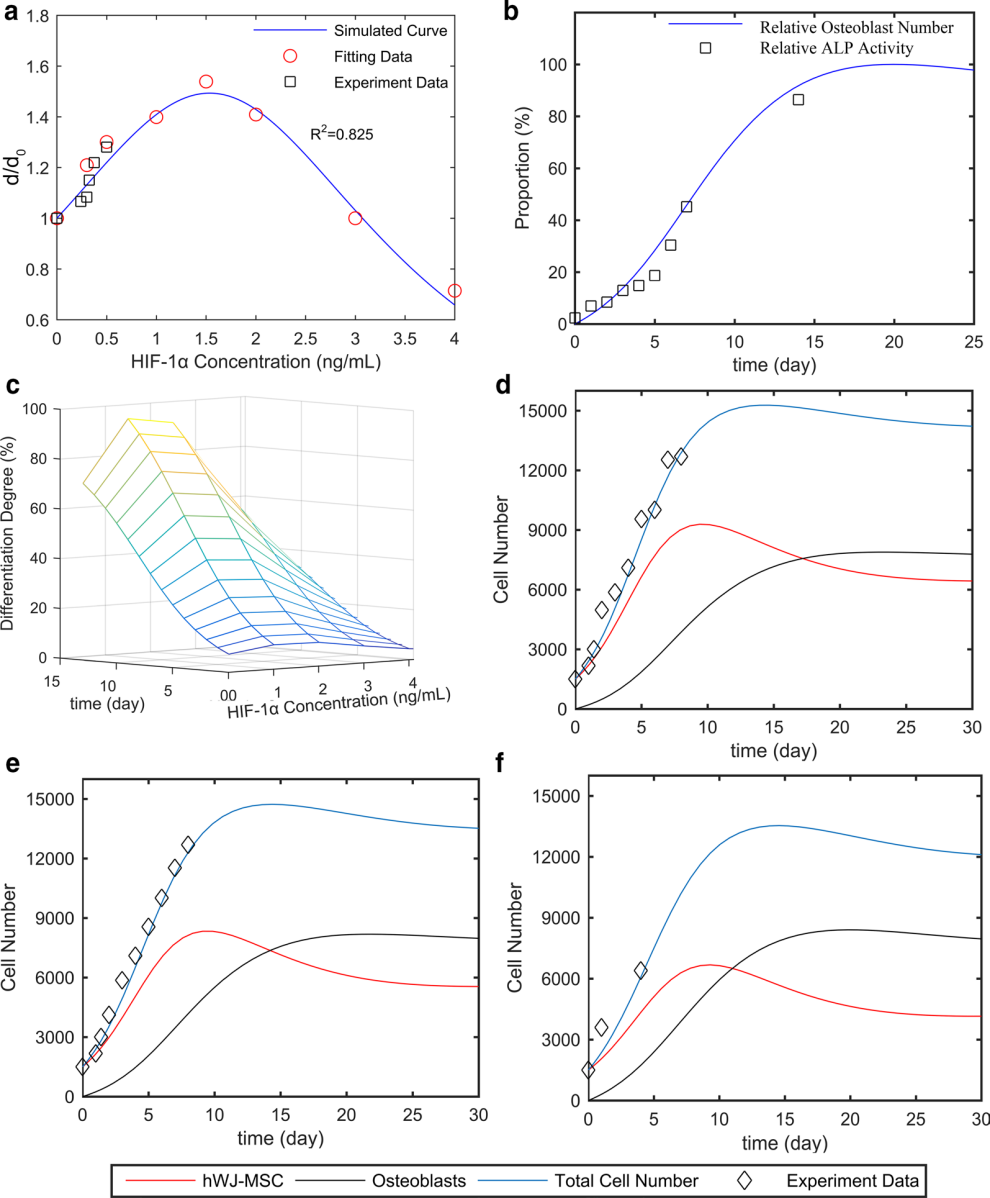
Two-stage cell-lineage models. hWJ-MSCs were incubated in a defined concentration of HIF-1α for the indicated times. **a** Relative differentiation rates of hWJ-MSCs at different concentrations of HIF-1α, **b** relative ALP activity (differentiation degrees of hWJ-MSCs) and relative osteoblast cells on different culture days. **c** Three-dimensional surface of differentiation degree evolving with time and HIF-1α. Cell number with an initial seeding density of 1500 cells per well (in a 96-well plate), as well as **d** 0, **e** 375, and **f** 1500 pg/mL HIF-1α. Abbreviations: *hWJ-MSCs* human umbilical cord Wharton’s jelly-derived mesenchymal stem cells, *HIF-1α* hypoxia-inducible factor 1α, *ALP* alkaline phosphatase

To quantitatively reveal the intrinsic connection between the concentration of HIF and osteogenic differentiation of hWJ-MSCs, 0–4000 pg/mL of HIF-1α was used to treat eight groups of hWJ-MSCs. A two-stage cell-lineage mathematical model was used to analyze the proliferation, apoptosis, and osteogenic differentiation rates of these hWJ-MSCs treated with different concentrations of HIF-1α. As shown in Fig. 8a, fitting data were employed to obtain the simulated formula ($\frac{d}{{d_{0} }} = \frac{1}{{0.14H^{2} - 0.43H + 1}}$) and curve (blue curve), showing that the differentiation rate first increased and then decreased with the increase in HIF concentration. More specifically, the differentiation rate reached a peak at 1500 pg/mL HIF-1α.

According to our study, the concentrations of HIF-1α produced by HUVECs in the control, m-HAP, np80, and np20 groups were 240, 300, 325, and 375 pg/mL, respectively. The black square represents the differentiation rate promoted by 240, 300, 325, and 375 pg/mL HIF-1α, and this matches well with the simulated curve. In addition, the differentiation degrees of the hWJ-MSCs (relative ALP activity) treated with different concentrations of HIF-1α increased similarly increased with time. Therefore, in order to simplify the model, we consider their growth patterns to be similar or even identical. We found that the increase in ALP activity from Day 0 to Day 7 was proportional to the number of osteoblasts, and osteoblast cells reached their peak at the platform period. Therefore, after fitting the ALP activity with the relative osteoblasts cell number curve (osteoblasts cell number / maximum osteoblasts cell number), the maximum of ALP activity was predicted and relative ALP activity (ALP activity/ maximum of ALP activity), representing the differentiation degree, was acquired (Fig. 8b). Combining these two studies, the three-dimensional surface of the differentiation degree evolving with time and HIF-1α was obtained (Fig. 8c).

In order to estimate the optimal culture time, it was necessary to simulate cell numbers. After elucidating the differentiation rate under different concentrations of HIF-1α, we simulated the size of each population. The simulation utilized an initial seeding density of 1500 cells per well (in a 96-well plate) using different concentrations of HIF-1α. As shown in Fig. 8d–f, the experimental data (black square) match well with the simulated total cell numbers (blue curve), which is a sum of the number of hWJ-MSCs and osteoblasts, and this supports the ability of this model to predict the number of hWJ-MSCs and osteoblasts at any time point. Moreover, the osteoblast cell number approached the platform period at 21, 18, and 15 days with 0, 375, and 1500 pg/mL HIF-1α, respectively. This model provides the optimum culture time for guiding tissue engineering, and it also provides direct evidence that HIF-1α accelerates the osteogenic differentiation of hWJ-MSCs.

## Discussion

With recent advances in nanobiomaterials, nano-based artificial bone substitutes have been an area of intense investigation. The accumulating evidence suggests that there are complex interactions between cells and nanobiomaterials due to their capacity to penetrate cell membranes and increase internal retention times [24, 25]. A previous study revealed that collagen/alginate nanofilms can adsorb onto the MSC membrane to activate intracellular signaling cascades and promote osteogenic differentiation [26]. Elegant experiments by Wu and his colleagues clearly demonstrated that TiO_2_ nanotubes can improve vascularization and osteogenic differentiation by facilitating paracrine effects and cell junctions via EC-MSC interactions [27]. For the purpose of developing excellent candidates for bone tissue engineering, it is necessary to clarify the direct crosstalk between nano-based bone substitutes and cells implicated in bone repair as well as their indirect interactions. However, our current understanding of this is still limited. In the present study, we utilized an indirect co-culture model to further elucidate the biological effects of HANPs on MSCs in regard to the indirect interactions mediated by ECs.

Cytotoxicity is a primary issue for assessing the biocompatibility of any nanobiomaterial. Although our previous study found that HANPs did not directly influence the viability or apoptosis of hWJ-MSCs, they may still exert different impacts via the mediation of other cells [28]. Thus, it was necessary to evaluate the cytotoxic effects of HANPs on hWJ-MSCs mediated by HUVECs. Interestingly, after incubation in CM for 24 h and 72 h, hWJ-MSC viability was maintained and even elevated in the 0–50 µg/mL HANP groups, especially in the np20 group, indicating the existence of effector molecules in the CM. When the concentration of HANPs reached 100 µg/mL, they became cytotoxic to the hWJ-MSCs. However, 0–100 µg/mL m-HAP had no influence on hWJ-MSC viability (Fig. 3). Jiang et al. have shown that engineered nanoparticles of a particular size can have distinct endocytic routes and kinetics associated with altered downstream signaling involved in regulating target cell functions [29]. In our previous study, we showed that np20 and np80 were endocytosed by HUVECs, and this was followed by morphologic changes and the appearance of large vacuoles, indicating the activated state of the HUVECs. Additionally, np20, with their faster uptake speed and increased accumulation, might result in a stronger activation of HUVECs, possibly resulting in increased hWJ-MSC viability via paracrine signaling. Conversely, few m-HAPs can be endocytosed by HUVECs, and this might account for their limited influence on the metabolism of hWJ-MSCs [9].

To further explore the potential osteoinductive effect of activated HUVECs, a subcytotoxic dose of 50 µg/mL HAPs was used in subsequent studies. The CM collected from the activated HUVECs promoted extracellular calcium deposition, ALP activity, and osteogenic proteins expression in hWJ-MSCs, as well as the mRNA expression of osteogenic genes (Figs. 4, 5). Runx2, an essential transcription factor involved in specifying the osteoblast lineage [30], showed a substantial enhancement in the np20 group, indicating a strong osteoinductive effect on hWJ-MSCs (Fig. 4a and Fig. 5c, d). The np20 group demonstrated a 1.5-fold improvement in COLI expression at Day 7 (Figs. 4b, 5c, d) and a double increase at Day 14, which implied the presence of additional differentiated osteoblasts in the HANP-treated groups (Fig. 4b) [30]. OCN is a mature stage bone marker [31], and this gene showed a significant increase in the HANP groups at Day 14 (Fig. 4c), indicating that np20 and np80 can accelerate bone maturation compared to m-HAP. ALP is an early marker of osteoblast differentiation, and it obviously increased with culture time in each group, especially the np20 group, revealing that additional transformation occurred from MSCs to osteoblasts (Fig. 4d). Pluripotency markers, NANOG, OCT3/4, and SOX2 imply the capacity for differentiation [32]. As shown in Fig. 4e–g, the decreased expression in the genes of the HANP groups implied that most of the hWJ-MSCs in HAP groups had transformed into osteoblasts.

Our data demonstrated that the endocytosis of HANPs by HUVECs was associated with an improved osteogenic differentiation of hWJ-MSCs. However, the cause of this outcome is currently unclear. In terms of the paracrine function of HUVECs, we focused on soluble differentiation-inducing proteins in the supernatant of activated HUVECs. HIF-1α signaling is essential in coupling ossification and angiogenesis during bone regeneration [33, 34]. Heikal et al. reported that injured ECs secrete more HIF-1α even under normoxia conditions [35]. It has also been shown that exposure to HANPs inhibits the angiogenic ability of HUVECs [9]. Thus, we measured the concentrations of HIF-1α in the CM, and the results showed that the HIF-1α content increased in the HANP treatment groups compared to the m-HAP and control groups (Fig. 6a). To identify the role of HIF-1α in the osteogenic differentiation of hWJ-MSCs, we used 2-MeOE2, which is a specific HIF-1α inhibitor, was used. The decreased concentration of HIF-1α paralleled the impaired mineralized matrix deposition and ALP activity in these hWJ-MSCs, indicating that HANPs can promote the HIF-1α production of HUVECs to facilitate the osteogenesis of hWJ-MSCs (Fig. 7).

To properly apply HANPs for use in bone tissue engineering, it is necessary to gain further insights into the mechanisms by which HANPs promote the osteogenic differentiation of hWJ-MSCs mediated by HUVECs. The ERK1/2 pathway is downstream of HIF-1α [36] and is fundamental to the differentiation of MSCs [37]. In this work, the concentrations of HIF-1α in the CM coincide well with the p-ERK1/2 levels in the hWJ-MSCs (Fig. 6b, c). When 2-MeOE2 was applied, the p-ERK1/2 expression in the hWJ-MSCs failed to be activated, indicating that HIF-1α functioned upstream of ERK1/2 signaling. To directly address the role of ERK1/2 signaling in the osteogenic differentiation of hWJ-MSCs, PD98059, a specific MEK inhibitor, was used. The suppression of ERK1/2 signaling resulted in the lowest osteogenic differentiation of hWJ-MSCs. One possible reason for this occurrence is that the ERK1/2 pathway plays a key role in both HIF-1α signaling and in the apoptosis and proliferation signaling pathways, which could be responsible for the observed changes in osteogenic differentiation in these cells [38, 39]. Additionally, this could also be related to the presence of vascular endothelial growth factor (VEGF). VEGF is one of the downstream effectors of HIF-1α signaling [33], and it can also promote the osteogenic differentiation of MSCs via activation of the ERK1/2 pathway [37]. Our previous study found that np20 induced the production of VEGF in HUVECs [9]; therefore, it is possible that the suppression of the ERK1/2 pathway may result in inhibition of VEGF, which would lead to the decreased osteogenic differentiation of hWJ-MSCs. According to the available experimental results, we can summarize as follows. HANPs are able to more optimally process better direct [5] and indirect osteoinductive effects than m-HAPs. Compared to autogenous bone grafts and bone allografts, there is an extensive source of HANP and without secondary damage and potential immunogenicity. However, compared to m-HAPs, HANPs can suppress the angiogenic ability of HUVECs [9] and exhibit slight cytotoxicity in both a time- and dose-dependent fashion.

Recently, growing evidence has demonstrated the importance of HIF-1α in the bone regeneration. However, few studies have been able to quantitatively predict the MSC differentiation rate under specific initial conditions, such as the HIF-1α concentration. Taking cell proliferation, apoptosis, and osteogenic differentiation into account, we present a mathematical model that combines a two-stage cell lineage with HIF-1α that is highly correlated with our experimental data. By fitting the differentiation rate of hWJ-MSCs in 0–4000 pg/mL HIF-1α, we acquired the equations for describing the differentiation rate, HIF-1α concentration, and time. As shown in Fig. 8d, this model can depict the cell number map under different HIF-1α concentrations, so that it is possible to explore the intrinsic dynamics of the two-stage system [40]. Additionally, this model mathematically validates the effect of HIF-1α on the osteogenic differentiation of hWJ-MSCs. Moreover, based on a multi-stage cell-lineage model and logistic model, our model is sufficiently stable to enable long-term predictions without falling into the trap of population unlimited explosion [41].

By using the existing experimental data, both the cell number and differentiation rate can be predicted with a defined initial cell seeding density and HIF-1α concentration. As such, the optimum incubation time is also obtained. Consequently, we can predict the optimum concentration of HIF-1α and determine the most optimal time for osteogenesis, which is important for efficient tissue engineering. A two-stage cell-lineage model is applicable for predicting the proliferation and differentiation of stem cells, which have two cell lineages. On this basis, the model founded on the initial conditions and existing experimental data can be established to identify the optimum culture conditions in vitro, which will assist in optimizing bone repair in vivo.

## Conclusion

**Fig. 9:**
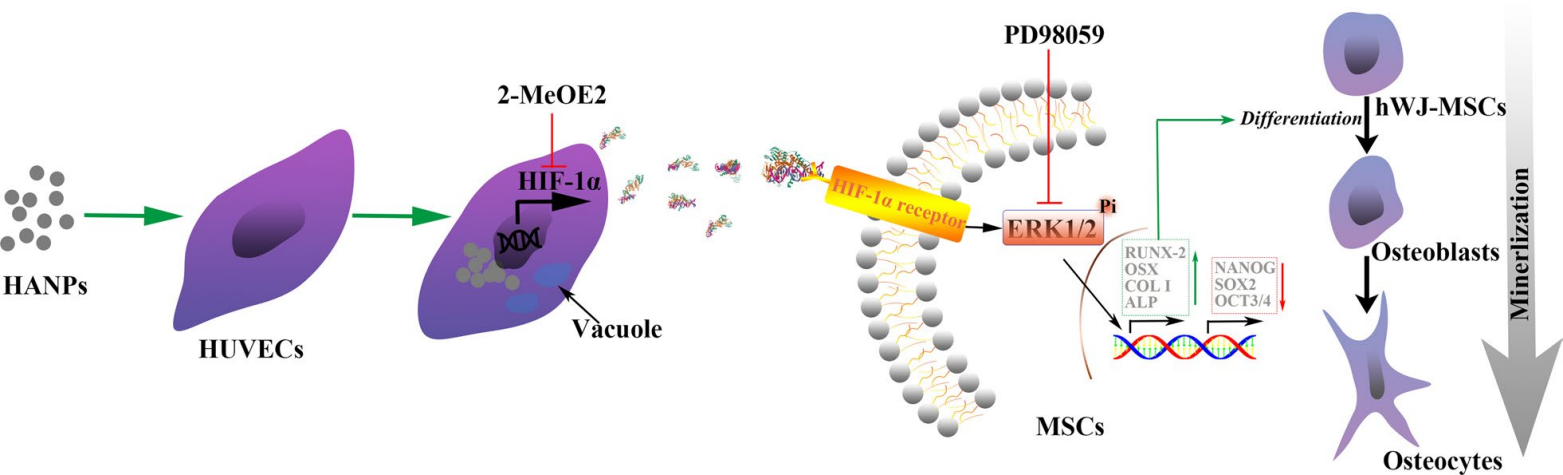
A schematic illustration of the possible mechanisms

In this study, we explored the specific biological effects of HANPs on hWJ-MSCs mediated by HUVECs. Compared to m-HAPs, both np20 and np80 showed slight cytotoxicity in both a time- and dose-dependent fashion. Importantly, the size of the HANPs appeared to have no significant impact on this cytotoxicity. Our data also showed that HANPs, especially np20, were capable of facilitating HUVECs to secrete increased levels of HIF-1α, which directly correlated with the enhanced osteogenic differentiation of hWJ-MSCs via the activation of the ERK1/2 pathway (Fig. 9). More remarkably, the results from the two-stage cell-lineage model suggested that HIF-1α exerted a dose-dependent stimulatory effect on the osteogenic differentiation rate of hWJ-MSCs. Additionally, the optimum concentration of HIF-1α and incubation time were estimated based on the initial conditions using an in vitro model, which could be invaluable in the future for tissue engineering applications. Collectively, these observations provide evidence that HANPs may improve bone regeneration by modulating cell–cell interactions.

## Supplementary Information

**Additional file 1:** HIF-1α concentration in CM at 18 and 24 h.

## Data Availability

The datasets used and analyzed during the current study are available from the corresponding author on reasonable request.

## Abbreviations

HA: Hydroxyapatite
HANPs: HA nanoparticles
hWJ-MSCs: Human umbilical cord Wharton’s jelly-derived mesenchymal stem cells
HUVECs: Human umbilical vein endothelial cell
m-HAP: Micro-sized HAP particles
PBS: Phosphate-buffered saline
ECM: EC medium
2-MeOE2: 2-Methoxyestradiol
ELISA: Enzyme-linked immunosorbent assay
RUNX-2: Runt-related transcription factor 2
Col I: Type I collagen
OCN: Osteocalcin
ALP: Alkaline phosphatase
SOX 2: SRY-related HMG-box 2
ERK: Extracellular signal-related kinases
VEGF: Vascular endothelial growth factor
