# Supplementary material for: Potential Osteoinductive Effects of Hydroxyapatite Nanoparticles on Mesenchymal Stem Cells by Endothelial Cell Interaction
Source: Nanoscale Res Lett. 2021 Apr 26;16:67. doi: 10.1186/s11671-021-03522-1 (PMC8076414; doi:10.1186/s11671-021-03522-1)
Supplement: Supplementary file 1 — Additional file 1: HIF-1α concentration in CM at 18 and 24 h. [file 11671_2021_3522_MOESM1_ESM.docx]

Table 1s

HIF-1α concentration in CM at 18 and 24 h.

| Incubation time | HIF-1α concentration (pg/mL) |
| --- | --- |
| 18 h | Con 266.9±31.7 np20 349.2±34.4  np80 292.9±57.9 mHAP 280.3±38.2 |
| 24 h | Con 312.1±51.8 np20 393.6±34.5  np80 332.5±31.4 mHAP 360±37.2 |
